# Health outcomes up to 3 years and post-exertional malaise in patients after hospitalization for COVID-19: a multicentre prospective cohort study (CO-FLOW)

**DOI:** 10.1016/j.lanepe.2025.101290

**Published:** 2025-04-06

**Authors:** Julia C. Berentschot, L. Martine Bek, Manon Drost, Rita J.G. van den Berg-Emons, Gert-Jan Braunstahl, Gerard M. Ribbers, Joachim G.J.V. Aerts, Merel E. Hellemons, Majanka H. Heijenbrok-Kal, CO-FLOW collaboration Group, Joachim G.J.V. Aerts, L. Martine Bek, Julia C. Berentschot, Rita J.G. van den Berg-Emons, Sieshem Bindraban, Wouter J.B. Blox, Jasper van Bommel, Shai A. Gajadin, Michel E. van Genderen, Diederik A.M.P.J. Gommers, Majanka H. Heijenbrok-Kal, Merel E. Hellemons, Roxane Heller, Erwin Ista, Stephanie van Loon-Kooij, Chantal Luijkx, Rutger Osterthun, Laurien Oswald, Gerard M. Ribbers, Ronald N. van Rossem, Herbert J. van de Sande, Robert van der Stoep, Janette J. Tazmi-Staal, Markus P.J.M. Wijffels, Eva G. Willems

**Affiliations:** a Department of Respiratory Medicine, Erasmus MC, University Medical Center Rotterdam, Rotterdam, the Netherlands; b Department of Rehabilitation Medicine, Erasmus MC, University Medical Center Rotterdam, Rotterdam, the Netherlands; c Department of Respiratory Medicine, Franciscus Gasthuis & Vlietland Hospital, Rotterdam, the Netherlands; d Rijndam Rehabilitation, Rotterdam, the Netherlands

**Keywords:** COVID-19, Long COVID, Long-term health outcomes, Post-exertional malaise

## Abstract

**Background:**

Many patients experience long-lasting health problems after COVID-19. The study aimed to assess 3-year trajectories of a comprehensive set of patient-reported outcome measures (PROMs) in patients hospitalized for COVID-19, particularly focusing on the 2- to 3-year trajectory. Additionally, we evaluated prevalence of post-exertional malaise (PEM) at 3 years, its risk factors, co-occurring health problems, and the 3-year trajectories of patients with and without PEM.

**Methods:**

The CO-FLOW multicentre prospective cohort study followed up adults hospitalized for COVID-19 in 7 hospitals, located in the Netherlands. Study assessments were performed at 3, 6, 12, 24, and 36 months post-discharge, conducted between July 1, 2020, and May 22, 2024. PROMs on recovery, symptoms, fatigue, mental health, cognition, participation, sleep quality, work status, health-related quality of life (HRQoL), and PEM were collected. Generalized estimating equations were used to assess health trajectories and multivariable logistic regression to identify risk factors for PEM.

**Findings:**

In total, 299/344 (87%) patients completed the 3-year follow-up and were included in the analysis. Complete recovery rates increased (p < 0.001), from 12% at 3 months to 24% at 3 years. Symptoms of impaired fitness, fatigue, and muscle weakness (all p < 0.0019) and PROMs for fatigue score, participation, return to work, and HRQoL (all p < 0.005) improved significantly over time, while PROMs for cognitive failures worsened (p < 0.001). Between the 2- and 3-year visits, memory problems (OR 1.4 [1.1–1.7], p < 0.001), and scores of fatigue (MD +1.0 [0.4–1.6], p = 0.002), cognitive failures (MD +2.2 [0.9–3.4], p < 0.001), and SF-36 mental component summary (−2.2 [−3.1 to −1.3], p < 0.001) significantly worsened. At 3 years, 66% of patients experienced fatigue, 63% impaired fitness, 59% memory problems, and 53% concentration problems. PROMs showed that 62% reported poor sleep quality, 55% fatigue, and 28% cognitive failures. PEM was reported by 105/292 (36%) patients at 3 years; risk factors were female sex (OR 3.4 [95% CI 1.9–6.0], p < 0.001), pre-existing pulmonary disease (3.0 [1.7–5.6], p < 0.001), physical inactivity pre-COVID-19 (2.3 [1.2–4.1], p = 0.008), and ICU treatment for COVID-19 (1.8 [1.02–3.0], p = 0.04). Concurrent fatigue, cognitive failures, and dyspnea were more common in patients with (42%) than without (6%) PEM. Patients with PEM showed poor health outcomes throughout the entire follow-up period, including worsening fatigue and HRQoL during the third year.

**Interpretation:**

Many health problems persisted up to 3 years post-discharge, with self-reported fatigue and cognitive problems worsening in the third year. PEM was common, and linked to a more severe phenotype of long COVID. These findings highlight the urgent need to optimize treatment options and investigate underlying pathological mechanisms of COVID-19.

**Funding:**

The 10.13039/501100001826Netherlands Organisation for Health Research and Development (ZonMw); Rijndam Rehabilitation; Laurens.

**Research in context**

**Evidence before this study**

We conducted a PubMed search for studies examining long-term health problems in patients previously hospitalized for COVID-19, including publications up to October 29, 2024, without language restrictions. The following search terms were used: (“COVID-19” OR “SARS-CoV-2” OR “Coronavirus disease 2019” or “long COVID”) AND “hospital∗” AND (“long-term∗” OR “follow∗” OR “recovery∗”) AND (“health outcomes” OR “patient-reported outcomes” OR “persistent” OR “sequelae”) AND (“2 year∗” OR “3 year∗”). Additionally, we searched PubMed for research articles on post-exertional malaise (PEM) in patients with long COVID using the terms: (“COVID-19” OR “SARS-CoV-2” OR “Coronavirus disease 2019” or “long COVID”) AND “post-exertional malaise”. The results indicated that only a limited number of studies have assessed health outcomes up to 3 years post-infection, reporting persistence of symptoms in most patients. Few studies have focused on PEM, but not in patients hospitalized for COVID-19, highlighting a notable gap in the current understanding of this condition within long COVID research.

**Added value of this study**

In this multicentre prospective cohort study with follow-up up to 3 years after hospitalization for COVID-19, the findings showed that patient experienced persisting and even worsening of several health issues up to 3 years. Specifically, while some health outcomes improved over time, self-reported fatigue and cognitive problems worsened between the second and third year. Similarly, joint pain, sleep disturbances, resuming work, and health-related quality of life (HRQoL) deteriorated during this period. Notably, only 24% of patients hospitalized for COVID-19 reported complete recovery at 3 years, underscoring the substantial health burden. This study also highlights post-exertional malaise (PEM), experienced by 36% of patients, with 98% of them also reporting co-occurring health issues. Patients with PEM showed significantly worse health outcomes throughout the 3-year follow-up period, suggesting that PEM may represent a more severe phenotype of long COVID. Risk factors for PEM included female sex, pre-existing pulmonary disease, and ICU admission, aligning with factors known to impact long-term COVID-19 recovery.

**Implications of all the available evidence**

The persistence and worsening of health problems in the third year after hospitalization for COVID-19 highlight the need for optimizing management strategies. The complexity and long-lasting health impact of COVID-19 remains poorly understood. PEM may serve as a marker for a more severe phenotype of long COVID. Early phenotyping of patients could improve risk stratification and enable more tailored aftercare, which may improve long-term outcomes. Additionally, our findings underscore the urgent need for research into the pathophysiology of long COVID and potential pharmacological treatments. Future studies should focus on identifying biomarkers and clinical tools to monitor symptom progression, ultimately enhancing management strategies and care for patients with long COVID.

## Introduction

Since December 2019, the World Health Organization has reported over 776 million confirmed cases of SARS-CoV-2,^1^ causing COVID-19, with a substantial number of these patients requiring hospitalization. The aftermath of COVID-19 showed that many patients do not recover to pre-COVID-19 health with persistent health problems for months or even years,^2^^,^^3^ a condition referred to as ‘long COVID’ or ‘Post COVID-19 Condition’.^4^^,^^5^

Long COVID is a heterogeneous disease, with patients experiencing a broad range of symptoms. The severity of these symptoms varies among patients, with fluctuations and relapses of symptoms over time being common.^6^ The most common symptoms include fatigue and cognitive problems. In addition, there is increasing awareness that post-exertional malaise (PEM) is a prevalent and debilitating symptom of long COVID. PEM is the abnormal worsening of symptoms after minimal physical or cognitive activity occurring immediately or delayed by hours or days after the activity.^7^^,^^8^ PEM limits daily activities and reduces health-related quality of life (HRQoL).^9^ Approximately 55–89% of the patients with long COVID experience PEM.^10^, ^11^, ^12^, ^13^ Although PEM is recognized as a debilitating feature of long COVID, it has been scarcely evaluated—particularly in patients who had been hospitalized for COVID-19,—leaving significant gaps in understanding its long-term prevalence, risk factors, and prognosis.^14^

Hospitalization for COVID-19 is considered a risk factor for developing long COVID. It is estimated that during the early phase of the pandemic, 50–70% of patients hospitalized for COVID-19 experience long COVID, compared to 7–30% of non-hospitalized patients, after 12 or more weeks post-infection.^15^, ^16^, ^17^ Studies have shown that, although some symptoms gradually decreased over time, many patients reported persistent symptoms up to 2 years after hospitalization.^2^^,^^3^^,^^18^

Insights into health outcomes up to 3 years after hospitalization for COVID-19 remain scarce. Two studies compared health outcomes between 2 and 3 years after hospitalization, showing persistence of symptoms at 3 years in 40–54% of patients.^19^^,^^20^ Likewise, a healthcare database study demonstrated substantial residual risk and health burden of long COVID in the third year after hospitalization.^21^ Longitudinal and more comprehensive assessments are largely lacking, although such insights are warranted to better understand the long-term health outcomes. Gaining insights into the health outcomes over time can assist policy makers and healthcare providers in refining COVID-19 aftercare strategies and guidelines.

The primary study aim was to assess trajectories of patient-reported health outcomes up to 3 years after hospitalization for COVID-19, with a particular focus on changes between the second and third year. The second aim was to assess the prevalence of PEM at 3 years post-discharge, evaluate co-occurring health problems, explore its risk factors, including demographic and clinical characteristics, and assess the 3-year trajectories of patients with and without PEM.

## Methods

### Study design and participants

This study is part of the “COvid-19 Follow-up care paths and Long-term Outcomes Within the Dutch health care system” (CO-FLOW) study, a prospective multicentre cohort study that follows up patients discharged from hospitals in the Rotterdam-Rijnmond-Delft region in the Netherlands. The CO-FLOW study protocol has been described in detail elsewhere.^22^ The study was performed in 7 hospitals (1 academic and 6 regional hospitals) and 3 rehabilitation centres (1 medical rehabilitation centre and 2 skilled nursing facilities). The study included patients between July 2020 and October 2021 who had been hospitalized for COVID-19, aged ≥ 18 years, had sufficient knowledge of the Dutch or English language, and were within 6 months post-discharge. Diagnosis of COVID-19 was based on either positive reverse transcription polymerase chain reaction or a clinical diagnosis (symptoms and chest radiological abnormalities) combined with positive serology for COVID-19. Incapacitated patients (e.g., dementia) were not included. Eligible patients were informed about the CO-FLOW study at hospital discharge and were recruited during routine follow-up at the outpatient clinic of one of the participating centres or during their inpatient stay in a rehabilitation centre. In the Netherlands, it was standard practice to offer post-discharge follow-up at the outpatient clinic of the discharging hospital to patients hospitalized for COVID-19, with the first visit generally scheduled 6–12 weeks post-discharge. Patients attending this visit were invited to participate in the study. Data on the total eligible recruitment population was unknown, but recruitment of study participants occurred independently of the patient’s recovery status and primarily depended on availability of research personnel. Study visits were performed at 3, 6, 12, and 24 months after hospital discharge.

The CO-FLOW study was extended to further monitor the trajectories of health outcomes by administering a survey of patient-reported outcome measures (PROMs) at 3 years post-discharge. The Medical Ethics Committee of the Erasmus MC, University Medical Center Rotterdam, approved the CO-FLOW study (MEC-2020-0487) and its extension. The study has been registered in the International Clinical Trial Registry Platform (NL8710). Participants provided written informed consent before the start of study measurements. Those participating in the extended CO-FLOW study provided new written informed consent. In this study, we included patients that filled in the survey at 3 years.

### Procedures

Procedures of the CO-FLOW study visits up to 2 years are described in detail elsewhere.^22^ In short, study visits comprised a comprehensive assessment of objectively assessed physical and cognitive functioning and a symptom questionnaire. Additionally, a survey was sent via e-mail or postal mail alongside each visit. Baseline characteristics and routine follow-up data regarding pulmonary and radiological outcomes were retrospectively collected from medical records at the participating facilities and during the study visits. At the end of the 2-year follow-up period, study participants received an information letter about continuing follow-up by email or postal mail. Patients involved in the extended study were followed up with a survey at 3 years post-discharge. The follow-up survey included PROMs that assessed health problems persisting at the 2-year visit. Additionally, we added the DePaul Symptom Questionnaire to assess PEM and the Work Limitations Questionnaire (WLQ) to assess work limitations. All collected data were stored in the Castor Electronic Data Capture system (Castor EDC, Amsterdam, the Netherlands).

### Baseline characteristics

Characteristics included patients’ age, sex, body mass index (BMI), migration background, education level, employment status, smoking status, and comorbidities at hospital admission. Pre-COVID-19 leisure time physical activity was assessed with the Saltin–Grimby Physical Activity Level Scale questionnaire.^23^ In-hospital characteristics included treatment for COVID-19, thrombosis, delirium, type of oxygen support, intensive care unit (ICU) admission, and the length of stay (LOS) in both ICU and hospital. Patients were classified based on their discharge date to reflect the timing of COVID-19 waves: first wave (Feb–Jun 2020), second wave (Jul 2020–Jan 2021), and third wave (Feb–Jun 2021).

### Study outcome measurements

#### Recovery

Self-reported recovery status from COVID-19, as compared to pre-COVID-19 health status, was assessed with the Core Outcome Measure for self-reported recovery from COVID-19 and dichotomized into completely recovered and not completely recovered (mostly recovered, somewhat recovered, half recovered, and not recovered at all).^24^ Additionally, patients rated their recovery status from COVID-19 on a numeric scale from 0% to 100% at the 3-year follow-up.

#### Symptoms

New symptoms or worsened symptoms since the SARS-CoV-2 infection were assessed using a symptom questionnaire (Corona Symptom Checklist, 26 symptoms).^25^ At the 2- and 3-year follow-up, patients were asked to also rate the severity (mild, moderate, severe, or very severe) of these symptoms.

#### Patient-reported outcome measures (PROMs)

PEM was assessed using a modified version of the DePaul Symptom Questionnaire, five symptoms were rated for frequency (0–4) and severity (0–4) on a 5-point Likert scale with higher scores indicating greater severity. PEM was indicated if both were scored ≥2 for one or more symptoms, and a total sum score was calculated by summing the scores for both frequency and severity of each symptom (0–40) as severity measure.^26^^,^^27^ Fatigue was assessed with the Fatigue Assessment Scale (scores 0–50, cutoff ≥ 22)^28^; dyspnea with the Modified Medical Research Council (mMRC) Dyspnea Scale^29^; anxiety and depression with the Hospital Anxiety and Depression Scale, subscales Anxiety and Depression (subscale scores 0–21, cutoff ≥ 11)^30^; cognitive failures with the Cognitive Failures Questionnaire (CFQ, scores 0–100, cutoff > 43)^31^^,^^32^; sleep quality with the Pittsburgh Sleep Quality Index (scores 0–21, cutoff ≥ 5)^33^; participation in daily life activities with the Utrecht Scale for Evaluation of Rehabilitation-Participation (USER-P) on three scales: frequency, restrictions, and satisfaction (subscale scores 0–100)^34^; employment status with the iMTA Productivity Cost Questionnaire (categorized into no, partial, or full return to work) for patients with a paid job before SARS-CoV-2 infection^35^; work limitations due to health problems following COVID-19 with the Work Limitation Questionnaire on four scales: time management, physical, mental-interpersonal, and output demands (scores 0 [limited none of the time] - 100 [limited all the time])^36^, ^37^, ^38^; health-related quality of life with the 5-level EuroQoL-5D (EQ-5D-5L) questionnaire^39^ and the 36-item Short Form Health Survey (SF-36).^40^ The EQ-5D-5L consists of the 5-level EQ-5D index (0 [death] - 1 [perfect health]; negative scores indicate a health status worse than death) and a visual analogue scale (EQ-VAS, scores 0–100). The SF-36 consists of 8 domains (scores 0–100) and a physical (PCS) and mental component summary (MCS) score (T-scores with mean 50 and standard deviation 10).^41^

### Statistical analysis

Data are presented as mean with standard deviation (SD), median with interquartile range (IQR), or as number with percentage. Normality of data was checked with the Shapiro–Wilk test. Group comparisons for continuous variables were performed with the Mann–Whitney U test and for categorical variables with the Chi-squared test. To assess the trajectories of health outcomes over time, Generalized Estimating Equations (GEE) analyses for repeated measurements were used, with linear models for continuous outcomes and logistic models for dichotomous outcomes. The GEE is a semi-parametric approach which considers within- and between subject correlations and uses all available measurements despite incomplete data. We entered follow-up time (3, 6, 12, 24, and 36 months) as a fixed factor in the GEE analysis for the total cohort. Each GEE analysis was performed using an unstructured correlation matrix.

Multivariable logistic regression analysis with backward elimination of variables was performed to assess risk factors for PEM at 3 years. These variables included demographics and acute COVID-19 characteristics at hospital admission and were selected a priori, including age (years), sex (male or female), migration background (European or non-European), education (low, middle, or high), employment status (employed, unemployed, or retired), smoking status (never or current/former), physical activity level (inactive/light or moderate/vigorous), obesity (obese if BMI ≥ 30 kg/m^2^, yes/no), cardiovascular disease (yes/no), pulmonary disease (yes/no), diabetes (yes/no), COVID-19 wave (first, second, or third), steroid or anti-inflammatory treatment (yes/no), ICU admission (yes/no), and the LOS in hospital (days). The prevalence of PEM and co-occurring health problems (fatigue, cognitive failures, and dyspnea) in patients with PEM at 3 years were calculated using the cut-off scores of the questionnaires. Moreover, we compared the 3-year trajectories of total scores of fatigue, cognitive failures, and HRQoL (SF-36 PCS and MCS) between patients with and without PEM at 3 years using GEE analysis, correcting for demographics and acute COVID-19 characteristics at hospital admission that significantly (p < 0.05) differed between groups. Missing data in the variables included in the multivariable analyses were not imputed (missingness ≤ 1% per variable). A p value < 0.05 was considered statistically significant, unless stated otherwise. A Bonferroni-corrected α threshold was applied to correct for multiple comparisons in symptoms (α = 0.0019) and PROMs (α = 0.00417). All statistical analyses were performed with IBM SPSS Statistics version 28 (SPSS Inc., Chicago, IL, USA).

### Role of the funding source

The funders of the study had no role in study design, data collection, data analysis, data interpretation, or writing of the report.

## Results

### Study participants

**Fig. 1:**
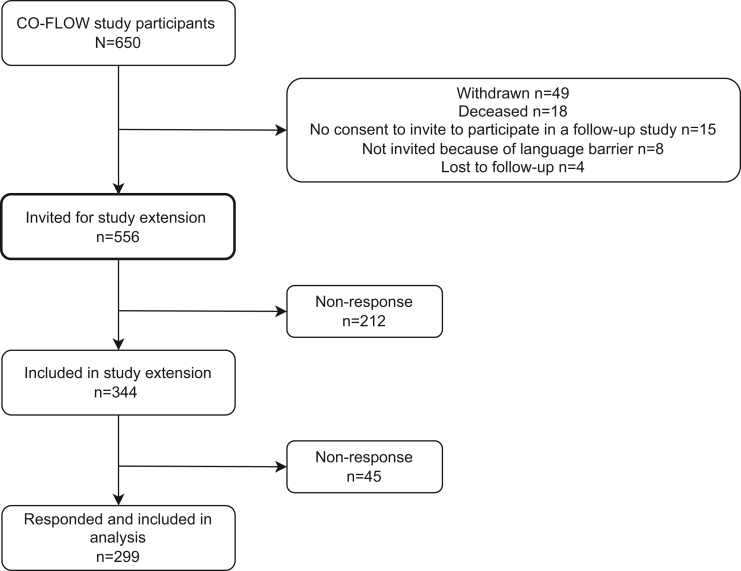
**Flowchart of participants included in analysis**.

Between July 1, 2020, and Sept 9, 2021, the CO-FLOW study prospectively enrolled 650 patients who had been hospitalized for COVID-19. After the 2-year follow-up period, 344/650 (53%) patients consented to participate in the CO-FLOW extension study with yearly follow-up survey, of whom 299/344 (87%) completed the 3-year survey and were included in the final analysis (Fig. 1). These patients were discharged from the hospital between March 26, 2020 and May 21, 2021. Table 1 presents the baseline characteristics of participants in- and excluded in the final study analysis. The latter group comprised patients (n = 306) who did not participate in the study extension and those (n = 45) who participated but lacked data at the 3-year visit. Patients included in the analysis were particularly characterized by older age (62 [55–69] years vs. 59 [50–67] years), a European migration background (87% vs. 57%), a high education level (35% vs. 25%), and a hospital admission during the first COVID-19 wave (33% vs. 23%), and had less frequently diabetes (13% vs. 26%) (all p ≤ 0.007) compared to those not participating; other characteristics were comparable between groups. Outcomes of recovery, symptoms, and PROMs at the 2-year visit did not differ significantly between patients in- and excluded in the analysis, except for the proportion of patients fully returning to work which was significantly lower in those included in the final analysis (62% vs. 84%, p < 0.001) (Supplementary Table S1).

**Table 1:** Comparison of baseline characteristics at hospital admission between CO-FLOW study participants included and not included in final analysis.

	N^a^	Participants included in analysis	N^a^	Participants not included in analysis	p value
**Patient characteristics**					
Age, years	299	62 (55–69)	351	59 (50─67)	<0.001
Sex, male	299	210 (70%)	351	239 (68%)	0.56
BMI, kg/m^2^	280	29 (26–32)	309	29 (26─33)	0.06
Migration background	297		333		<0.001
European		258 (87%)		191 (57%)	
Dutch Caribbean		25 (8%)		64 (19%)	
Asian		9 (3%)		30 (9%)	
Turkish		4 (1%)		24 (7%)	
(North) African		1 (1%)		24 (7%)	
Education^b^	296		329		0.007
Low		89 (30%)		133 (40%)	
Middle		104 (35%)		114 (35%)	
High		103 (35%)		82 (25%)	
Employment	296		331		0.06
Unemployed		37 (13%)		63 (19%)	
Employed		179 (61%)		193 (58%)	
Retired		80 (27%)		75 (23%)	
Smoking status	297		334		0.09
Never		118 (40%)		162 (49%)	
Former		173 (58%)		166 (50%)	
Current		6 (2%)		6 (1%)	
Physical activity level^c^	297		327		0.09
Inactive		30 (10%)		56 (17%)	
Light		164 (55%)		168 (51%)	
Moderate		85 (29%)		83 (25%)	
Vigorous		18 (6%)		20 (6%)	
Comorbidities	299		351		0.08
0		59 (20%)		57 (16%)	
1		88 (29%)		85 (24%)	
≥2		152 (51%)		209 (60%)	
Obesity (BMI ≥ 30 kg/m^2^)		111 (37%)		155 (44%)	0.07
Diabetes		39 (13%)		91 (26%)	<0.001
Cardiovascular disease/hypertension		108 (36%)		149 (43%)	0.10
Pulmonary disease		72 (24%)		90 (26%)	0.65
Renal disease		29 (10%)		30 (9%)	0.61
Gastrointestinal disease		16 (5%)		15 (4%)	0.52
Neuromuscular disease		33 (11%)		35 (10%)	0.66
Malignancy		36 (12%)		33 (9%)	0.28
Autoimmune/inflammatory disease		32 (11%)		36 (10%)	0.85
Mental disorder		12 (4%)		20 (6%)	0.32
**In-hospital characteristics**					
Vaccinated before admission	299		347		0.13
Yes		295 (99%)		1 (1%)	
No		4 (1%)		346 (99%)	
COVID-19 wave^d^	299		351		0.005
First		100 (33%)		80 (23%)	
Second		138 (46%)		201 (57%)	
Third		61 (20%)		70 (20%)	
Chest x-ray abnormalities	283		336		0.64
Normal		32 (11%)		35 (10%)	
Moderate		57 (20%)		78 (23%)	
Severe		194 (69%)		223 (66%)	
COVID-19 directed treatment	299		351		
None		67 (22%)		67 (19%)	0.30
(Hydroxy)chloroquine		9 (3%)		3 (1%)	0.04
Steroids		205 (69%)		251 (72%)	0.41
Antivirals		35 (12%)		62 (18%)	0.03
Anti-inflammatory		37 (12%)		39 (11%)	0.62
Convalescent plasma		5 (2%)		3 (1%)	0.35
Thrombosis	298	81 (27%)	350	84 (24%)	0.22
Delirium	298	81 (27%)	350	84 (24%)	0.22
Requiring oxygen supplementation	299	290 (97%)	351	337 (96%)	0.50
Requiring high flow nasal cannula	299	98 (33%)	349	110 (32%)	0.82
ICU admission	299	129 (43%)	351	144 (41%)	0.59
Invasive mechanical ventilation	299	118 (40%)	351	117 (33%)	0.11
Length of intubation, days	113	16 (8–29)	116	13 (9─27)	0.56
Tracheostomy	298	44 (15%)	350	46 (13%)	0.12
Length of ICU stay, days	127	20 (10–33)	144	15 (8─30)	0.04
Length of hospital stay, days	299	14 (6–31)	351	10 (5─26)	0.04
**Time interval from discharge to follow-up questionnaires, days**					
Three months	232	98 (91–107)	222	95 (88─105)	
Six months	285	189 (182–200)	242	185 (180─194)	
One year	286	371 (364–379)	224	367 (362─378)	
Two years	285	735 (729–746)	180	731 (725─740)	
Three years	297	1100 (1091–1128)	NA	NA	

Data are presented as median (interquartile range) or n (%) at time of hospital admission. The following variables were dichotomized for statistical analysis: migration background was categorized as European vs. non-European groups combined, smoking status as never vs. former/current, physical activity level as inactive/light vs. moderate/vigorous, and treatment as no treatment vs. any received treatment. BMI, Body Mass Index; SARS-CoV-2, Severe Acute Respiratory Syndrome Coronavirus 2; ICU, Intensive Care Unit; NA, Not Applicable.

a Number of patients with available data for each variable.

b Education comprises low (primary or secondary education); middle (high school); high (postsecondary education or university).

c Pre-COVID-19 leisure time physical activity level was measured with the Saltin–Grimby Physical Activity Level Scale questionnaire.^23^

d Patients were classified by discharge date: the first COVID-19 wave (Feb–Jun 2020), second wave (Jul 2020–Jan 2021), and third wave (Feb–Jun 2021).^42^

### Recovery status

Overall, the proportion of patients that felt completely recovered increased over time (p < 0.001) (Supplementary Table S2). The proportion of patients that felt completely recovered did not differ significantly between the 2- and 3- year visits (mean difference [MD] +0.02 [95% CI −0.03 to 0.06], p = 0.48). At 3 years, 72/297 (24%) of the patients felt completely recovered from COVID-19. Patients reported an average a recovery level of 80% (IQR 60–95) compared to their pre-COVID-19 health status.

### Symptoms

**Fig. 2:**
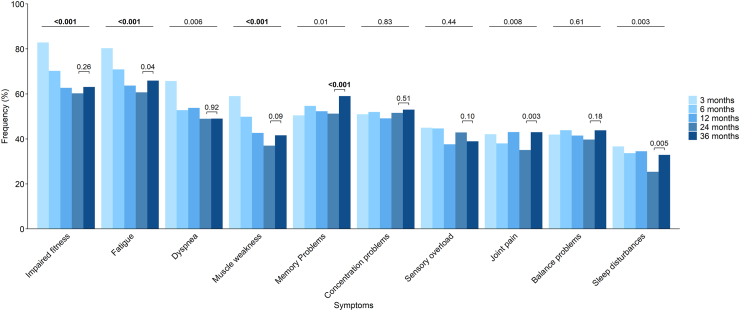
**Trajectories of the ten most prevalent symptoms in patients with COVID-19 up to 3 years after hospital discharge.** P values are obtained from Generalized Estimating Equations analysis, and are presented for changes over the overall follow-up period at the top and specifically from 2 to 3 years follow-up above the columns. A p value less than 0.0019 was considered statistically significant and is indicated in bold.

The prevalence of symptoms of impaired fitness, fatigue, and muscle weakness decreased significantly over total follow-up time (all p < 0.0019); a similar trend was found for dyspnea and sleep disturbances but these differences were not significant after Bonferroni correction (Fig. 2). Between the 2- and 3-year visits, the prevalence of memory problems significantly increased (odds ratio [OR] 1.4 [95% CI 1.1–1.7], p < 0.001); a similar trend was found for joint pain and sleep disturbances. The prevalence of concentration problems, sensory overload, and balance problems did not significantly change over time.

At 3 years, more than half of the patients experienced fatigue (197/299 [66%]), impaired fitness (188/298 [63%]), memory problems (176/298 [59%]), or concentration problems (158/298 [53%]) (Supplementary Table S3). Regarding the severity of these symptoms, impaired fitness was indicated as severe or very severe by 54/184 (28%) patients, fatigue by 71/196 (37%), memory problems by 36/175 (20%), and concentration problems by 41/157 (26%) at 3 years (Supplementary Table S4A and B).

### PROMs

**Fig. 3:**
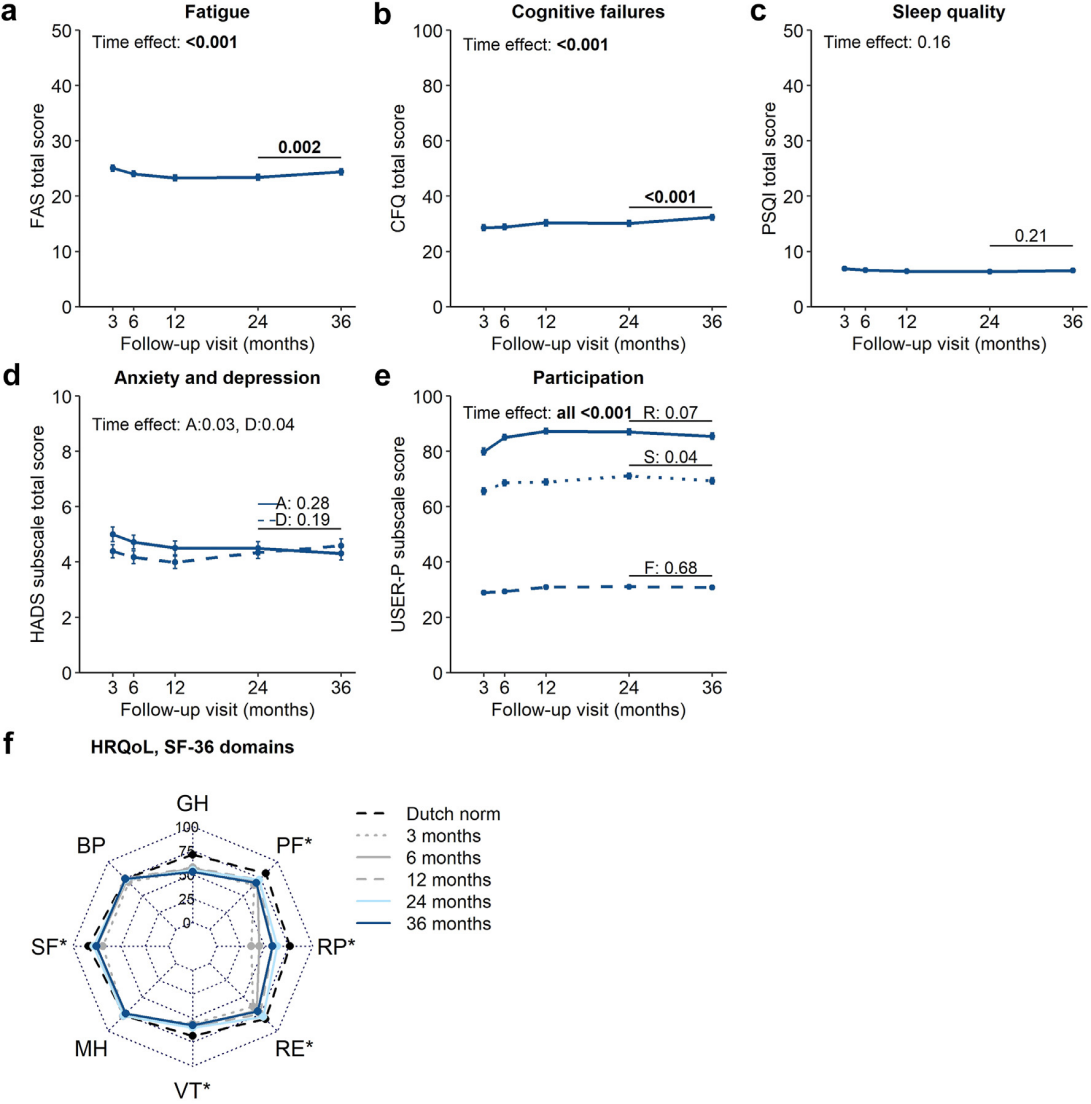
**Trajectories of PROMs in patients up to 3 years after hospitalization for COVID-19.** Data are presented as estimated means with standard errors obtained from Generalized Estimating Equations analysis. The trajectories from 3 to 36 months post-discharge are presented for health outcomes of fatigue (a), cognitive failures (b), sleep quality (c), anxiety [denoted by A] and depression [D] (d), and participation (e). In panel e, USER-P includes the subscales restriction [R], satisfaction [S], and frequency [F]. In the panels a–e, p values are presented for the overall time effect from 3 to 36 months (top left corner) and specifically for the trajectory between the 2- and 3-year visits (above trajectory line). A p value less than 0.00417 was considered statistically significant and is indicated in bold. For HRQoL, data are presented for each SF-36 domain in a spider plot (f), ∗ indicates a p value < 0.00417 for the trajectory between the 2- and 3-year visits. FAS, Fatigue Assessment Scale; CFQ, Cognitive Failures Questionnaire; PSQI, Pittsburgh Sleep Quality Index; HADS, Hospital Anxiety and Depression Scale; USER-P, Utrecht Scale for Evaluation of Rehabilitation-Participation; SF-36, 36-item Short Form Health Survey with the domains: GH, General Health; PF, Physical Functioning; RP, Physical Role Impairment; RE, Emotional Role Impairment; VT, Vitality; MH, Mental Health; SF, Social Functioning; BP, Bodily Pain.

Outcomes for fatigue, participation, return to work, and HRQoL improved significantly over time (all p < 0.005); a similar trend was found for mental health outcomes, but these differences did not reach significance after Bonferroni correction (Fig. 3 and Supplementary Table S5). Cognitive failures significantly worsened over time (p < 0.001). Between the 2- and 3-year visits, the fatigue score (MD +1.0 [95% CI 0.4–1.6], p = 0.002), cognitive failures score (+2.2 [0.9–3.4], p < 0.001), and SF-36-MCS score (−2.2 [−3.1 to −1.3], p < 0.001) significantly worsened; a similar trend was found for the participation satisfaction component score, and employment status. The sleep quality score did not significantly change over time.

At 3 years, 176/286 (62%) patients experienced poor sleep quality, 163/295 (55%) fatigue, 80/283 (28%) cognitive failures, 33/295 (11%) depression, and 25/295 (9%) anxiety (Supplementary Table S5). Among patients with a paid job before their SARS-CoV-2 infection, 57/129 (44%) had not fully returned to work. Regarding work limitations due to COVID-19, patients (n = 121) were limited for a median of 20.0% (IQR 0.0–45.0) of their time in time management demands, 83.3% (70.3–100.0) in physical demands, 19.4% (2.8–41.7) in mental-interpersonal demands, and 20.0% (0.0–45.0) in output demands. Patients had a median 7.7% loss in productivity compared to reference values of healthy (not limited) employees. Regarding HRQoL, patients reached a median SF-36 physical component score of 47.7 (34.9–54.0) and mental component score of 50.0 (39.5–55.5) at 3 years.

### Post-exertional malaise (PEM)

**Fig. 4:**
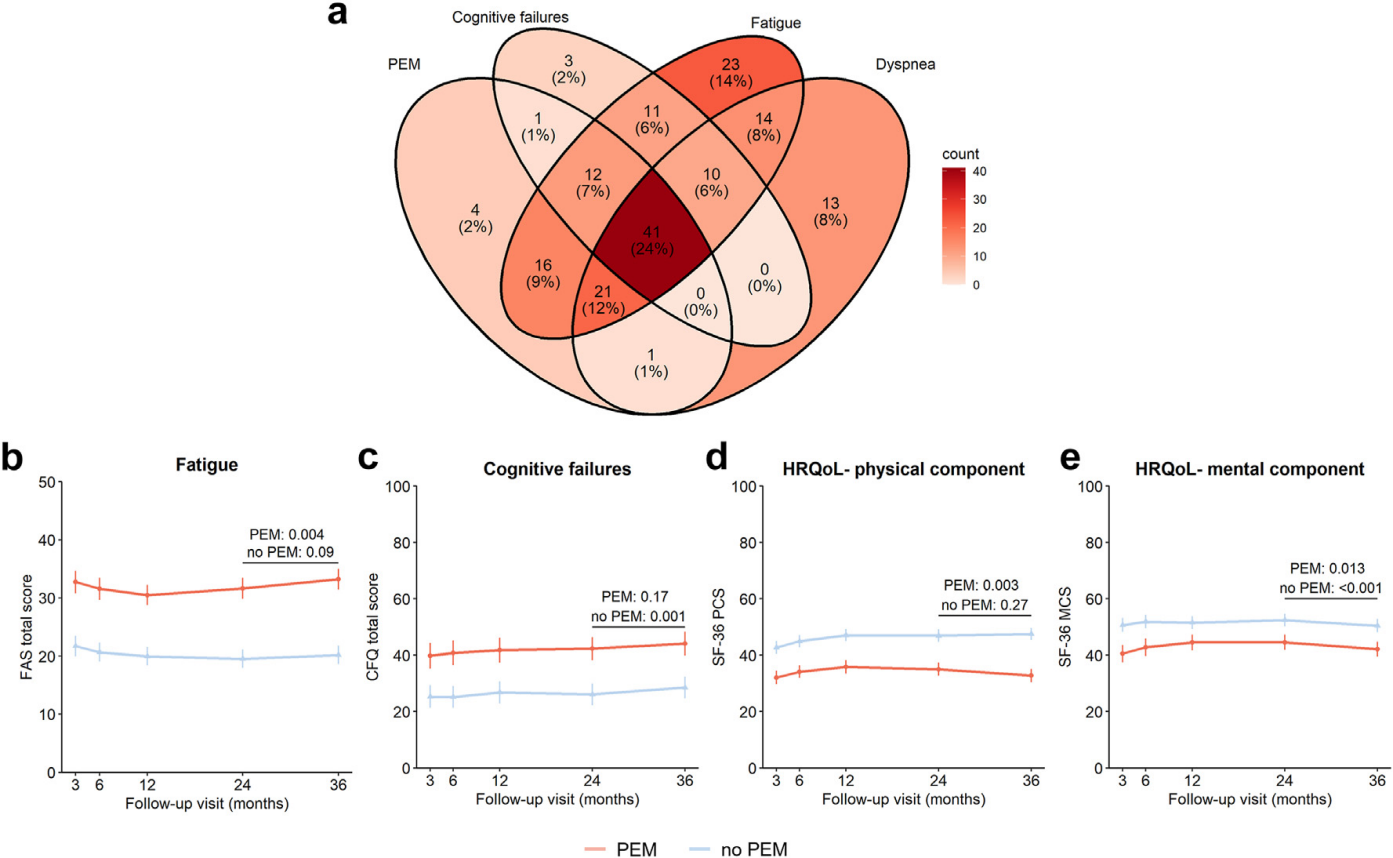
**Co-occurring health problems and health outcome trajectories in patients with COVID-19 with and without PEM at 3 years.** Co-occurring health problems in patients with PEM (a) at 3 years after hospital discharge. Group comparisons were performed for the 3-year trajectories of fatigue (b), cognitive failures (c), and HRQoL physical component (d) and mental component summary (e) scores, adjusted for sex, pre-COVID-19 employment, pre-COVID-19 education level, pre-COVID-19 physical activity level, pre-existing comorbidities obesity, pulmonary disease, and cardiovascular disease, and intensive care unit admission during hospitalization. P values are presented for within group differences between the 2- and 3-year visit, obtained from Generalized Estimating Equations analysis. PEM, Post-Exertional Malaise; FAS, Fatigue Assessment Scale; CFQ, Cognitive Failures Questionnaire; HRQoL, Health-Related Quality of Life; SF-36, 36-item Short Form Health Survey; PCS, Physical Component Summary; MCS, Mental Component Summary.

A total of 105/292 (36%) patients reported PEM at 3 years. These patients were particularly characterized by a higher proportion of females (48% vs. 19%, p < 0.001), having ≥1 comorbidities (89% vs. 76%, p = 0.009), and more frequently physical inactive pre-COVID-19 (20% vs. 5%, p < 0.001), compared to those without PEM (Supplementary Table S6). In the PEM group, the median PEM total score was 18/40 (13–23). Many patients with PEM (92/95 [98%]) experienced co-occurring health problems, with 41/95 (42%) patients experiencing concurrent fatigue, cognitive failures, and dyspnea (Fig. 4a). Among patients without PEM, 10/179 (6%) also experienced these overlapping health problems. Moreover, patients with PEM reported an average recovery level of 55% compared to 88% among those without PEM at 3 years. The frequency and severity of PEM symptoms are shown in Supplementary Table S7.

Risk factors for PEM after hospitalization for COVID-19 included female sex (OR 3.4 [95% CI 1.9–6.0], p < 0.001), pre-existing pulmonary disease (3.0 [1.7–5.6], p < 0.001), pre-COVID-19 physical inactivity (2.3 [1.2–4.1], p = 0.008), and ICU treatment for COVID-19 (1.8 [1.02–3.0], p = 0.04), and a trend was found for younger age (0.97 [0.946–1.001], p = 0.061).

Fig. 4b–e presents the 3-year trajectories of fatigue, cognitive failures, and HRQoL in patients with and without PEM. Patients with PEM showed worse outcomes over the entire 3-year follow-up period compared to patients without PEM, with the fatigue score (MD +1.6 [95% CI 0.50–2.7], p = 0.004), SF-36-PCS (−2.3 [−3.7 to −0.75], p = 0.003), and SF-36-MCS (−2.4 [−4.3 to −0.51], p = 0.013) scores worsening significantly between the 2- and 3-year visits. In patients without PEM, the cognitive failures score (+2.4 [0.94–3.9], p = 0.001) and SF-36-MCS (−2.0 [−3.0 to −1.0], p < 0.001) worsened significantly in the third year.

## Discussion

This multicentre cohort study showed that, despite improvements over time, many health problems persisted up to 3 years after hospitalization for COVID-19. Fatigue and cognitive problems were most frequently reported throughout follow-up, and even worsened between the 2- and 3-year assessments. Furthermore, joint pain, sleep disturbances, resuming work, and HRQoL components, showed a similarly worsening trend. At 3 years, only 24% of our patients reported complete recovery. Importantly, 36% of patients experienced PEM, with most of these patients (98%) experiencing co-occurring health problems. Patients with PEM at 3 years showed poor health outcomes over the entire 3-year follow-up period. Female sex, pre-existing pulmonary diseases, and ICU admission for COVID-19 were identified as risk factors for PEM.

Although fatigue and cognitive failures showed statistically significant worsening between 2 and 3 years of follow-up, the changes in these outcome scores for the total group were relatively small. Taken into account the standard minimal clinically important difference (MCID) of 4 points on the FAS score,^28^ 25% of patients reported a clinically important worsening in fatigue scores. As the MCID has not been previously determined for the CFQ, a 10% change (10 points) in CFQ scores was used as the threshold. Between 2 and 3 years, 20% of patients reported a clinically important worsening in cognitive failures scores. On the contrary, 14% of patients had a clinically important improvement in fatigue scores and 10% in cognitive failures scores. Zhang and colleagues also found a worsening of symptoms (fatigue or muscle weakness, joint pain, and hair loss) from 2 to 3 years post-hospitalization for COVID-19, but their sample was less severely ill (4% ICU admission).^20^ Yang and colleagues observed no significant changes in symptom rates, while fatigue tended to improve, between 2 and 3 years after hospitalization for COVID-19.^19^ The discrepancies in findings across studies may be due to varying study designs, COVID-19 subpopulations, and assessment tools, requiring further cohort studies to confirm previous findings. Nonetheless, the overall picture remains that many problems not only persist over time, but some may even worsen. Additionally, symptoms of long COVID can remit and relapse over time,^6^ highlighting the importance of long-term monitoring beyond 3 years post-hospitalization to better understand disease trajectory and identify factors contributing to long-term recovery. These insights may assist policy makers and healthcare providers in refining COVID-19 aftercare strategies, research agendas, and guidelines.

At 3 years, 36% of our patients experienced PEM, a hallmark feature in myalgic encephalomyelitis/chronic fatigue syndrome (ME/CFS), and a key symptom in the recent Long COVID definition.^6^ However, PEM is a poorly understood symptom of long COVID. A population-based study of patients after a positive SARS-CoV-2 test reported a prevalence of PEM of 23.2% in females and 17.8% in males up to 18 months post-infection,^43^ compared to a prevalence of PEM ranging from 59% to 89% up to 12 months post-infection in patients with long COVID.^10^, ^11^, ^12^ This study demonstrates for the first time that PEM is also a prominent feature in patients after hospitalization for COVID-19.

Our findings show that females, patients with pre-existing pulmonary disease, patients with low pre-COVID-19 physical activity level, and ICU-treated patients for COVID-19 were more likely to experience PEM. These risk factors are also associated with other health problems following COVID-19,^3^^,^^44^^,^^45^ suggesting they contribute to a broader risk for long COVID rather than being linked to just one specific symptom. Notably, 42% of patients with PEM experienced concurrent fatigue, cognitive failures, and dyspnea, while this was only 6% in those without PEM. Moreover, patients with PEM at 3 years showed poorer health outcomes throughout the entire study period. Previous studies on PEM following COVID-19 showed worsening of symptoms following different types of activities,^46^ long-term persistence of PEM,^47^ and its association with co-occurring health problems^10^^,^^11^^,^^43^^,^^47^ and a poorer disease course.^10^ Together, these findings support the idea that patients with PEM represent a more severe phenotype of long COVID with potentially unfavourable prognosis. We suggest that the early identification of PEM is crucial to optimize individualized care and improve long-term outcomes.

Long COVID is a complex condition characterized by a wide range of symptoms. Our findings extend and add to findings from previous studies showing persistent symptoms lasting up to 3 years after hospitalization for COVID-19,^19^, ^20^, ^21^ with the prevalence and burden of health outcomes fluctuating over time. Although hospitalization for COVID-19 is a significant risk factor for developing long COVID,^45^^,^^48^ it can develop after any severity of acute COVID-19 illness. Long COVID is linked to new-onset conditions such as dysautonomia, particularly postural orthostatic tachycardia syndrome (POTS), and ME/CFS. Several hypotheses have been proposed to underlie mechanisms of long COVID. These include viral persistence or latent viral reactivation, immune dysregulation, neuroinflammation, mitochondrial dysfunction, autoimmunity, and microvascular thrombosis, which have also been specifically linked to fatigue and cognitive sequelae.^49^, ^50^, ^51^, ^52^ Ideally, future studies should include comprehensive assessment of long-term health outcomes, including PEM, dysautonomia, and POTS, and underlying mechanisms of long COVID, to capture its full spectrum.

The strengths of this study include its longitudinal multicentre design with a 3-year follow-up period assessing a comprehensive set of PROMs in a large and clinically well-defined cohort. This approach enabled detailed health assessment at five time points following hospital discharge, providing a thorough evaluation of health trajectories. Recently, PEM has gained recognition as a debilitating symptom of long COVID,^43^ however, studies assessing PEM remain scarce.^14^ Our study addresses this gap by being among the first to assess PEM in patients previously hospitalized for COVID-19, providing insights into its prevalence and its role as a defining feature of long COVID. The study also has limitations, including the absence of control groups of individuals without COVID-19 and individuals not hospitalized for COVID-19, and the inability to compare our outcomes with pre-COVID-19 levels. Our findings may not generalize to non-hospitalized patients or those vaccinated before admission, as vaccination has consistently been shown to lower the risk of long-term health problems.^53^ This study lacks data on the eligible recruitment population due to the surge of patients admitted to the participating centres. However, recruitment of study participants occurred independently of the patient’s recovery status and primarily depended on availability of research personnel. Moreover, our patient characteristics align with those of the average Dutch patients hospitalized for COVID-19.^54^ The CO-FLOW study composed of a high percentage of ICU-treated patients (42%) which may contribute to the relatively high percentage of patients with prolonged symptoms compared to other studies on patients hospitalized for COVID-19. Selection bias may have played a role in this long-term follow-up cohort, as 47% (306/650) of patients did not participate in the study extension, potentially leading to an overrepresentation of individuals with long-term health problems. However, although patients included in the 3-year follow-up analysis differed slightly in baseline characteristics from those not included (dropouts and non-responders), health outcomes at 2 years did not differ significantly between the two groups. The study assessment relied on PROMs, which, while providing valuable insight into patients’ experience of symptoms and their impact on daily functioning, may introduce bias in estimating health problems due to their inherently subjective nature. To enhance the validity of our findings, validated and widely recognized questionnaires were used to measure health outcomes. The CO-FLOW study comprised both objective and self-reported assessments up to 2 years post-hospitalization; objective assessments indicated generally good physical recovery and minimal cognitive deficits. However, the high symptomatic burden in self-reports was often not reflected in objective measures. To reduce the burden of repeated assessments, a survey was conducted in the extended study at 3 years. Notably, to determine whether changes in health outcomes are reflected in both self-reports and objective measurements, future studies may consider to include a comprehensive evaluation of both self-reported and objective outcomes. Further, PEM was only assessed at the 3-year follow-up, lacking a longitudinal evaluation, and relied on the DePaul Symptom Questionnaire as opposed to the gold standard but burdensome invasive two-day cardiopulmonary exercise testing.

In summary, health problems remained prevalent in patients up to 3 years after hospitalization for COVID-19, with a worsening of self-reported fatigue and cognitive problems during the third year post-discharge. At 3-year follow-up, 36% of patients experienced PEM, with many also experiencing co-occurring health problems, representing a more severe phenotype of long COVID. Our findings highlight the urgent need for research into effective management strategies for long COVID, as well as the importance of ongoing monitoring of disease trajectory to better understand the long-term outcomes of COVID-19.

## Contributors

JB and LB shared first authorship and MH and MHK shared senior authorship, contributing equally to this paper. All authors were involved in the study design and had full access to the data in the study. All authors and members of the CO-FLOW Collaboration Group contributed to the acquisition, analysis, or interpretation of data. JB, LB, MD, RBE, MH, and MHK drafted the manuscript. JB, LB, MD, and MHK performed the statistical analysis and verified the data. All authors and members of the CO-FLOW Collaboration Group critically revised and approved the manuscript. MHK, MH, and RBE provided supervision and had the final responsibility for the decision to submit for publication.

## Data sharing statement

Deidentified participant data will be made available on reasonable request to the corresponding author. The study protocol has been previously published.

## Declaration of interests

The authors declare that they have no competing interests related to this paper.
