# Supplementary material for: Health outcomes up to 3 years and post-exertional malaise in patients after hospitalization for COVID-19: a multicentre prospective cohort study (CO-FLOW)
Source: Lancet Reg Health Eur. 2025 Apr 6;53:101290. doi: 10.1016/j.lanepe.2025.101290 (PMC12237789; doi:10.1016/j.lanepe.2025.101290)
Supplement: Supplementary Tables S1–S7 [file mmc1.docx]

# Supplemental materials

**Health outcomes up to 3 years and post-exertional malaise in patients after hospitalization for COVID-19: a multicentre prospective cohort study (CO-FLOW)**

Julia C. Berentschot^1*^, MSc, L. Martine Bek^2*^, MSc, Manon Drost^1^, MD, Rita J.G. van den Berg-Emons^2^, PhD, Gert-Jan Braunstahl^1,3^, PhD, Gerard M. Ribbers^2,4^, PhD, Joachim G.J.V. Aerts^1^, PhD, Merel E. Hellemons^1**^, PhD, Majanka H. Heijenbrok-Kal^2,4**^**,** PhD, on behalf of the CO-FLOW collaboration Group^***^

^*^ Authors share first authorship; ^**^ Authors share senior authorship

**Affiliations:**

^1^Department of Respiratory Medicine, Erasmus MC, University Medical Center Rotterdam, Rotterdam, The Netherlands.

^2^Department of Rehabilitation Medicine, Erasmus MC, University Medical Center Rotterdam, Rotterdam, The Netherlands.

^3^Department of Respiratory Medicine, Franciscus Gasthuis & Vlietland Hospital, Rotterdam, The Netherlands.

^4^Rijndam Rehabilitation, Rotterdam, The Netherlands.

**Contents**

|  |  |
| --- | --- |
| **Table S1.** Comparison of recovery status, symptoms, and PROMs between CO-FLOW study participants in- and excluded in the final analysis. | **P2** |
| **Table S2.** Trajectory of recovery status in COVID-19 patients up to 3 years after hospital discharge. | **P4** |
| **Table S3.** Trajectories of symptoms in patients with COVID-19 up to 3 years after hospital discharge. | **P5** |
| **Table S4A.** The severity of the ten most prevalent symptoms in COVID-19 patients at 2 and 3 years after hospital discharge. | **P6** |
| **Table S4B.** Categorical outcomes on the mMRC dyspnea scale in COVID-19 patients up to 3 years after hospital discharge. | **P6** |
| **Table S5.** Trajectories of validated PROMs in COVID-19 patients up to 3 years after hospital discharge. | **P7** |
| **Table S6.** Comparison of baseline characteristics at hospital admission between participants with and without PEM at 3 years post-discharge. | **P9** |
| **Table S7.** The frequency and severity of PEM symptoms in patients at 3 years after hospitalization for COVID-19. | **P11** |

**Tables**

**Table S1.** Comparison of recovery status, symptoms, and PROMs at 2 years post-discharge between CO-FLOW study participants included and not included in the final analysis.

|  | **Participants included in final analysis**  **N=299** | **Participants not included in final analysis**  **N=351** | **P value** |
| --- | --- | --- | --- |
| **Recovery status, *n*** | *266* | *177* | 0.89 |
| Completely recovered | 72 (27%) | 49 (28%) |  |
| Not completely recovered | 194 (73%) | 128 (72%) |  |
| **Symptoms** |  |  |  |
| Impaired fitness | 176/292 (60%) | 135/210 (64%) | 0.36 |
| Fatigue | 173/285 (61%) | 129/208 (62%) | 0.77 |
| Dyspnea | 140/286 (49%) | 111/207 (54%) | 0.31 |
| Muscle weakness | 108/292 (37%) | 81/211 (38%) | 0.75 |
| Memory problems | 149/291 (51%) | 125/211 (59%) | 0.07 |
| Concentration problems | 150/291 (52%) | 109/211 (52%) | 0.98 |
| Sensory overload | 123/287 (43%) | 73/208 (35%) | 0.08 |
| Joint pain | 102/291 (35%) | 68/210 (32%) | 0.53 |
| Balance problems/dizziness | 116/292 (40%) | 84/210 (40%) | 0.95 |
| Sleep disturbances | 74/292 (25%) | 67/211 (32%) | 0.11 |
| **PROMs** |  |  |  |
| **Fatigue, *n*** | *279* | *156* |  |
| FAS, total score | 23.2 (9.0) | 24.3 (8.9) | 0.24 |
| Fatigue (FAS ≥22) | 139 (50%) | 84 (54%) | 0.42 |
| **mMRC dyspnea scale, *n*** | *284* | *182* |  |
| mMRC ≥1 | 105 (37%) | 78 (43%) | 0.21 |
| **Mental health, *n*** | *283* | *164* |  |
| HADS-A, total score | 4.5 (4.1) | 4.8 (4.3) | 0.39 |
| Anxiety (HADS-A ≥11) | 25 (9%) | 18 (11%) | 0.46 |
| HADS-D, total score | 4.3 (3.9) | 5.0 (4.0) | 0.14 |
| Depression (HADS-D ≥11) | 25 (9%) | 21 (13%) | 0.18 |
| **Cognition, *n*** | *278* | *162* |  |
| CFQ, total score | 30.0 (17.6) | 31.2 (19.7) | 0.69 |
| Cognitive failures (CFQ >43) | 61 (22%) | 42 (26%) | 0.34 |
| **Sleep quality, *n*** | *276* | *153* |  |
| PSQI, total score | 6.3 (4.2) | 6.8 (4.3) | 0.29 |
| Poor sleep quality (PSQI ≥5) | 166 (60%) | 95 (62%) | 0.69 |
| **Participation, *n*** | *280* | *158* |  |
| USER-P, Frequency | 31.2 (10.1) | 28.7 (11.7) | 0.03 |
| USER-P, Restrictions | 87.4 (17.0) | 85.3 (19.6) | 0.22 |
| USER-P, Satisfaction | 71.5 (17.5) | 67.2 (20.7) | 0.06 |
| **Employment status, n** | *146* | *88* | **<0.001** |
| IPCQ, not or partially returned to work | 55 (37%) | 14 (16%) |  |
| iPCQ, fully returned to work | 91 (62%) | 74 (84%) |  |
| **HRQoL** |  |  |  |
| *EQ-5D-5L, n* | *282* | *158* |  |
| Index value | 0.80 (0.21) | 0.78 (0.22) | 0.18 |
| EQ-VAS | 74.2 (18.3) | 72.2 (17.9) | 0.18 |
| *SF-36, n* | *282* | *152* |  |
| Physical component summary | 44.8 (10.9) | 44.4 (10.1) | 0.51 |
| Mental component summary | 49.8 (9.8) | 48.3 (10.6) | 0.11 |

The data comprise raw test outcomes and are presented as mean (standard deviation), n/N (%), or n (%). Participants not included in the 3-year follow-up consist of 306 patients who did not participate in the study extension and 45 patients who participated but lacked data at 3 years. Employment status is presented for patients with a paid job pre-COVID-19. P values are obtained using Mann-Whitney U test or Chi-squared test as appropriate, a P value less than 0.00417 was considered statistically significant and is indicated in bold. PROMs, Patient-Reported Outcome Measures; FAS, Fatigue Assessment Scale; mMRC, Modified Medical Research Council dyspnea scale; HADS-A, Hospital Anxiety and Depression Scale-subscale Anxiety; HADS-D, Hospital Anxiety and Depression Scale-subscale Depression; CFQ, Cognitive Failures Questionnaire; PSQI, Pittsburgh Sleep Quality Index; USER-P, Utrecht Scale for Evaluation of Rehabilitation-Participation; iPCQ, iMTA Productivity Cost Questionnaire; HRQoL, Health-Related Quality of Life; EQ-5D-5L, 5-level EuroQoL-5D; EQ-VAS, EQ-Visual Analogue Scale; SF-36, 36-item Short Form Health Survey.

**Table S2.** Trajectory of recovery status in patients with COVID-19 up to 3 years after hospital discharge.

|  | **3 months** | **6 months** | **1 year** | **2 years** | **3 years** | **Time**  **P value** | **Comparison 2-3y**  **P value** |
| --- | --- | --- | --- | --- | --- | --- | --- |
| **Recovery status , *n*** | *78* | *157* | *246* | *266* | *297* |  |  |
| Completely recovered | 9 (12%) | 24 (15%) | 53 (22%) | 72 (27%) | 72 (24%) | <0.001 | 0.48 |
| Not completely recovered | 69 (88%) | 133 (85%) | 193 (78%) | 194 (73%) | 225 (76%) |  |  |
| *Categorical outcomes* |  |  |  |  |  |  |  |
| Not recovered at all | 0 (0%) | 0 (0%) | 0 (0%) | 6 (2%) | 15 (5%) |  |  |
| Somewhat recovered | 13 (17%) | 8 (5%) | 9 (4%) | 12 (5%) | 26 (9%) |  |  |
| Half recovered | 18 (23%) | 36 (23%) | 40 (16%) | 42 (16%) | 62 (21%) |  |  |
| Mostly recovered | 38 (49%) | 89 (57%) | 144 (59%) | 134 (50%) | 122 (41%) |  |  |
| Completely recovered | 9 (12%) | 24 (15%) | 53 (22%) | 72 (27%) | 72 (24%) |  |  |

Data are presented as n (%). Recovery status after COVID-19 was dichotomized into completely recovered or not completely recovered (mostly recovered, somewhat recovered, half recovered, and not recovered at all) for statistical analysis. P values are obtained from Generalized Estimating Equations analysis.

**Table S3.** Trajectories of symptoms in patients with COVID-19 up to 3 years after hospital discharge.

|  | **3 months** | **6 months** | **1 year** | **2 years** | **3 years** | **Time**  **P value** | **Comparison 2-3y**  **P value** |
| --- | --- | --- | --- | --- | --- | --- | --- |
| **Symptoms** |  |  |  |  |  |  |  |
| ≥ 1 | 202/211 (96%) | 264/278 (95%) | 263/286 (92%) | 252/292 (86%) | 266/296 (90%) | **<0.001** | 0.05 |
| Impaired fitness | 174/210 (83%) | 196/279 (70%) | 180/287 (63%) | 176/292 (60%) | 188/298 (63%) | **<0.001** | 0.26 |
| Fatigue | 57/71 (80%) | 90/127 (71%) | 128/201 (64%) | 173/285 (61%) | 197/299 (66%) | **<0.001** | 0.04 |
| Dyspnea | 46/70 (66%) | 66/125 (53%) | 107/199 (54%) | 140/286 (49%) | 146/298 (49%) | 0.006 | 0.92 |
| Muscle weakness | 124/210 (59%) | 139/279 (50%) | 122/286 (43%) | 108/292 (37%) | 124/298 (42%) | **<0.001** | 0.09 |
| Memory problems | 106/210 (50%) | 153/280 (55%) | 150/287 (52%) | 149/291 (51%) | 176/298 (59%) | 0.01 | **<0.001** |
| Concentration problems | 107/210 (51%) | 145/279 (52%) | 141/287 (49%) | 150/291 (52%) | 158/298 (53%) | 0.83 | 0.51 |
| Sensory overload | 27/60 (45%) | 54/121 (45%) | 76/202 (38%) | 123/287 (43%) | 116/298 (39%) | 0.44 | 0.10 |
| Joint pain | 88/209 (42%) | 106/279 (38%) | 123/286 (43%) | 102/291 (35%) | 128/298 (43%) | 0.008 | 0.003 |
| Balance problems/dizziness | 88/210 (42%) | 122/278 (44%) | 119/287 (41%) | 116/292 (40%) | 131/299 (44%) | 0.61 | 0.18 |
| Tingling/numbness in extremities | 79/219 (36%) | 91/277 (33%) | 99/286 (35%) | 90/288 (31%) | 98/298 (33%) | 0.72 | 0.58 |
| Hair loss | 78/210 (37%) | 55/279 (20%) | 30/287 (10%) | 35/290 (12%) | 58/296 (20%) | **<0.001** | **<0.001** |
| Sleep disturbances | 77/210 (37%) | 94/279 (34%) | 99/287 (34%) | 74/292 (25%) | 98/298 (33%) | 0.003 | 0.005 |
| Headache | 19/60 (32%) | 36/125 (29%) | 38/199 (19%) | 51/284 (18%) | 56/298 (19%) | 0.24 | 0.79 |
| Cough | 61/210 (29%) | 59/278 (21%) | 62/287 (22%) | 75/292 (26%) | 93/299 (31%) | 0.002 | 0.03 |
| Chest pain | 14/58 (24%) | 18/125 (14%) | 35/201 (17%) | 49/286 (17%) | 44/298 (15%) | 0.17 | 0.24 |
| Skin rash | 56/210 (27%) | 74/278 (27%) | 72/287 (25%) | 69/292 (24%) | 81/298 (27%) | 0.66 | 0.17 |
| Phlegm | 49/209 (23%) | 58/279 (21%) | 61/287 (21%) | 59/292 (20%) | 62/299 (21%) | 0.91 | 0.75 |
| Vision problems | 55/209 (26%) | 74/279 (27%) | 79/287 (28%) | 86/291 (30%) | 77/299 (26%) | 0.46 | 0.10 |
| Anosmia | 41/210 (20%) | 53/279 (19%) | 52/287 (18%) | 62/291 (21%) | 60/299 (20%) | 0.46 | 0.45 |
| Hoarseness | 50/210 (24%) | 68/278 (24%) | 63/287 (22%) | 60/291 (21%) | 59/299 (20%) | 0.30 | 0.74 |
| Ageusia | 41/210 (20%) | 55/278 (20%) | 59/287 (21%) | 56/292 (19%) | 62/299 (21%) | 0.95 | 0.42 |
| Stool problems | 34/210 (16%) | 54/279 (19%) | 45/287 (16%) | 44/292 (15%) | 63/299 (21%) | 0.05 | 0.005 |
| Hearing problems | 27/210 (13%) | 40/279 (14%) | 57/287 (20%) | 59/233 (20%) | 60/299 (20%) | 0.01 | 0.90 |
| Anxiety/nightmares | 32/210 (15%) | 41/280 (15%) | 39/287 (14%) | 30/292 (10%) | 33/298 (11%) | 0.12 | 0.66 |
| Claudication | 28/209 (13%) | 29/279 (10%) | 18/287 (6%) | 19/292 (7%) | 44/298 (15%) | **<0.001** | **<0.001** |
| Miction problems | 20/209 (10%) | 32/279 (11%) | 26/287 (9%) | 41/290 (14%) | 53/299 (18%) | 0.006 | 0.13 |
| The data comprise raw test outcomes and are presented as n/N (%). The differing denominators indicate missing data. The presence of symptoms was assessed with a symptom questionnaire (Corona Symptom Checklist, CSC) on new or worsened symptoms following SARS-CoV-2 infection. The symptoms fatigue, dyspnea, headache, chest pain, and sensory overload were added to the CSC in a later stage and therefore contain lower total numbers. P values are obtained from Generalized Estimating Equations analysis, a P value less than 0.0019 was considered statistically significant and is indicated in bold. | | | | | | | |

**Table S4A.** The severity of the ten most prevalent symptoms in patients with COVID-19 at 2 and 3 years after hospital discharge.

|  | **2 years** | | | | | **3 years** | | | | |
| --- | --- | --- | --- | --- | --- | --- | --- | --- | --- | --- |
| **Symptoms** | **N** | **Mild** | **Moderate** | **Severe** | **Very severe** | **N** | **Mild** | **Moderate** | **Severe** | **Very severe** |
| Impaired fitness | 149 | 34 (23%) | 65 (44%) | 42 (28%) | 8 (5%) | 184 | 45 (25%) | 85 (46%) | 49 (27%) | 5 (1%) |
| Fatigue | 152 | 24 (16%) | 66 (43%) | 48 (32%) | 14 (9%) | 196 | 42 (21%) | 83 (42%) | 58 (30%) | 13 (7%) |
| Muscle weakness | 89 | 19 (21%) | 43 (48%) | 24 (27%) | 3 (3%) | 124 | 33 (27%) | 64 (52%) | 24 (19%) | 3 (2%) |
| Memory problems | 128 | 36 (28%) | 41 (32%) | 44 (34%) | 7 (6%) | 175 | 55 (31%) | 84 (48%) | 32 (18%) | 4 (2%) |
| Concentration problems | 129 | 29 (23%) | 51 (40%) | 46 (36%) | 3 (2%) | 157 | 34 (22%) | 82 (52%) | 33 (21%) | 8 (5%) |
| Sensory overload | 111 | 30 (27%) | 39 (35%) | 35 (32%) | 7 (6%) | 115 | 23 (20%) | 61 (53%) | 29 (25%) | 2 (2%) |
| Balance problems /dizziness | 99 | 38 (38%) | 39 (39%) | 20 (20%) | 2 (2%) | 130 | 55 (42%) | 58 (45%) | 14 (11%) | 3 (2%) |
| Sleep disturbances | 65 | 14 (22%) | 18 (28%) | 31 (48%) | 2 (3%) | 98 | 21 (21%) | 48 (49%) | 25 (26%) | 4 (4%) |
| Joint pain | 85 | 24 (28%) | 35 (41%) | 23 (27%) | 3 (4%) | 128 | 31 (24%) | 70 (55%) | 25 (20%) | 2 (2%) |

Data are presented as n (%). At the 2-year and 3-year study visit, patients with symptoms were asked to indicate the severity of each symptom. The severity of the symptom dyspnea is not included as the severity of this symptom was assessed with the Modified Medical Research Council Dyspnea Scale (Table S4B).

**Table S4B.** Categorical outcomes on the mMRC dyspnea scale in patients with COVID-19 up to 3 years after hospital discharge.

|  | **3 months** | **6 months** | **1 year** | **2 years** | **3 years** |
| --- | --- | --- | --- | --- | --- |
| **mMRC dyspnea scale, *n*** | *220* | *283* | *281* | *284* | *296* |
| No dyspnea | 38 (17%) | 56 (20%) | 61 (22%) | 60 (21%) | 152 (51%) |
| Grade 0 | 98 (45%) | 123 (44%) | 119 (42%) | 117 (41%) | 34 (11%) |
| Grade 1 | 42 (19%) | 57 (20%) | 61 (22%) | 57 (20%) | 51 (17%) |
| Grade 2 | 37 (17%) | 39 (14%) | 34 (12%) | 39 (14%) | 35 (12%) |
| Grade 3 | 5 (2%) | 6 (2%) | 5 (2%) | 11 (4%) | 20 (7%) |
| Grade 4 | 0 (0%) | 2 (1%) | 1 (1%) | 0 (0%) | 4 (1%) |

Data are presented as n (%). The Modified Medical Research Council Dyspnea contains the following grades: grade 0, dyspnea only with strenuous exercise; grade 1, dyspnea when hurrying or walking up a slight hill; grade 2, walks slower than people of the same age because of dyspnea or has to stop for breath when walking at own pace; grade 3, stops for breath after walking 100 meters or after a few minutes; and grade 4, too dyspneic to leave the house or breathless when dressing.

**Table S5.** Trajectories of validated PROMs in patients with COVID-19 up to 3 years after hospital discharge.

|  | **3 months** | **6 months** | **1 year** | **2 years** | **3 years** | **Time**  **P value** | **Comparison 2-3y**  **P value** |
| --- | --- | --- | --- | --- | --- | --- | --- |
| **Fatigue, *n*** | *219* | *280* | *282* | *279* | *295* |  |  |
| FAS, total score | 24.9 (9.4) | 23.9 (8.9) | 23.4 (8.9) | 23.2 (9.0) | 24.3 (9.3) | **<0.001** | **0.002** |
| Fatigue (FAS ≥22) | 128 (58%) | 158 (56%) | 148 (53%) | 139 (50%) | 163 (55%) | 0.02 | 0.03 |
| **mMRC dyspnea scale, *n*** | *220* | *283* | *281* | *284* | *296* |  |  |
| mMRC ≥1 | 84 (38%) | 104 (37%) | 101 (36%) | 105 (37%) | 110 (37%) | 0.93 | 0.75 |
| **Mental health, *n*** | *217* | *278* | *282* | *283* | *295* |  |  |
| HADS-A, total score | 4.9 (4.2) | 4.7 (4.3) | 4.6 (4.4) | 4.5 (4.1) | 4.3 (4.1) | 0.03 | 0.28 |
| Anxiety (HADS-A ≥11) | 21 (10%) | 29 (10%) | 25 (9%) | 25 (9%) | 25 (9%) | 0.55 | 0.77 |
| HADS-D, total score | 4.2 (3.8) | 4.2 (3.9) | 4.0 (3.8) | 4.3 (3.9) | 4.5 (4.3) | 0.04 | 0.19 |
| Depression (HADS-D ≥11) | 18 (8%) | 21 (8%) | 23 (8%) | 25 (9%) | 33 (11%) | 0.35 | 0.24 |
| **Cognition, *n*** | *233* | *275* | *279* | *278* | *283* |  |  |
| CFQ, total score | 28.6 (18.1) | 28.8 (17.9) | 30.8 (18.3) | 30.0 (17.6) | 32.2 (18.1) | **<0.001** | **<0.001** |
| Cognitive failures (CFQ >43) | 44 (20%) | 62 (23%) | 62 (22%) | 61 (22%) | 80 (28%) | **0.002** | **0.001** |
| **Sleep quality, *n*** | *223* | *272* | *277* | *276* | *286* |  |  |
| PSQI, total score | 6.8 (4.0) | 6.6 (4.0) | 6.4 (4.0) | 6.3 (4.2) | 6.4 (4.0) | 0.16 | 0.21 |
| Poor sleep quality (PSQI ≥5) | 149 (67%) | 178 (65%) | 164 (59%) | 166 (60%) | 176 (62%) | 0.02 | 0.47 |
| **Participation, *n*** | *228* | *282* | *282* | *280* | *295* |  |  |
| USER-P, Frequency | 29.2 (10.4) | 29.5 (10.5) | 30.8 (10.3) | 31.2 (10.1) | 30.9 (10.7) | **<0.001** | 0.68 |
| USER-P, Restrictions | 80.4 (20.1) | 85.2 (16.8) | 87.2 (16.3) | 87.4 (17.0) | 85.4 (19.2) | **<0.001** | 0.07 |
| USER-P, Satisfaction | 66.5 (18.7) | 68.8 (18.6) | 68.8 (18.7) | 71.5 (17.5) | 69.5 (19.5) | **<0.001** | 0.04 |
| **Employment status, n** | *170* | *169* | *164* | *146* | *129* |  |  |
| IPCQ, not or partially returned to work | 128 (75%) | 95 (56%) | 63 (38%) | 55 (37%) | 57 (44%) | **<0.001** | 0.04 |
| iPCQ, fully returned to work | 42 (25%) | 74 (44%) | 101 (62%) | 91 (62%) | 72 (56%) |  |  |
| **HRQoL** |  |  |  |  |  |  |  |
| *EQ-5D-5L, n* | *222* | *280* | *283* | *282* | *295* |  |  |
| Pain and discomfort | 146 (66%) | 172 (61%) | 167 (59%) | 176 (62%) | 182 (62%) | 0.16 | 0.77 |
| Anxiety or depression | 69 (31%) | 84 (30%) | 76 (27%) | 79 (28%) | 92 (31%) | 0.16 | 0.20 |
| Mobility problem | 111 (50%) | 120 (43%) | 104 (37%) | 120 (43%) | 130 (44%) | **<0.001** | 0.71 |
| Personal care problem | 31 (14%) | 21 (8%) | 21 (8%) | 20 (7%) | 34 (12%) | 0.01 | 0.03 |
| Usual activity problem | 128 (58%) | 138 (49%) | 118 (42%) | 111 (39%) | 122 (41%) | **<0.001** | 0.39 |
| Index value | 0.75 (0.24) | 0.78 (0.22) | 0.80 (0.20) | 0.80 (0.21) | 0.79 (0.22) | **<0.001** | 0.10 |
| EQ-VAS | 69.2 (20.0) | 73.3 (20.0) | 74.1 (18.2) | 74.2 (18.3) | 75.2 (16.9) | **<0.001** | 0.17 |
| *SF-36, n* | *216* | *278* | *280* | *282* | *296* |  |  |
| Physical functioning | 66.1 (24.4) | 71.0 (22.6) | 73.0 (23.2) | 73.3 (24.1) | 69.3 (25.7) | **<0.001** | **<0.001** |
| Physical role impairment | 37.0 (42.2) | 45.0 (43.2) | 58.3 (43.1) | 63.0 (41.6) | 58.3 (44.4) | **<0.001** | 0.03 |
| Emotional role impairment | 65.6 (41.9) | 70.1 (39.9) | 74.3 (36.4) | 79.6 (34.1) | 71.4 (40.3) | **<0.001** | **<0.001** |
| Vitality | 56.3 (22.5) | 59.2 (22.4) | 59.6 (22.3) | 60.3 (22.6) | 57.5 (22.5) | **<0.001** | **<0.001** |
| Mental health | 76.7 (19.0) | 76.9 (18.5) | 77.8 (18.1) | 77.6 (17.1) | 74.3 (18.0) | **<0.001** | **<0.001** |
| Social functioning | 70.0 (28.0) | 75.9 (24.7) | 77.5 (24.6) | 78.6 (23.5) | 75.9 (24.3) | **<0.001** | 0.008 |
| Bodily pain | 69.8 (26.3) | 72.6 (25.9) | 75.0 (24.8) | 73.9 (25.2) | 74.9 (25.3) | 0.004 | 0.33 |
| General health | 54.7 (22.7) | 57.1 (22.5) | 57.2 (22.9) | 54.7 (23.2) | 52.9 (23.9) | **<0.001** | 0.03 |
| Physical component summary | 41.3 (10.4) | 43.3 (10.5) | 45.1 (10.5) | 44.8 (10.9) | 44.4 (11.5) | **<0.001** | 0.41 |
| Mental component summary | 48.0 (11.5) | 48.8 (11.6) | 49.0 (10.7) | 49.8 (9.8) | 47.5 (10.4) | **<0.001** | **<0.001** |

The data comprise raw test outcomes and are presented as mean (standard deviation) or n (%). Employment status is presented for patients with a paid job pre-COVID-19. P values are obtained from Generalized Estimating Equations analysis, a P value less than 0.005 was considered statistically significant and is indicated in bold. ICU, Intensive Care Unit; PEM, Post-Exertional Malaise; FAS, Fatigue Assessment Scale; mMRC, Modified Medical Research Council dyspnea scale; HADS-A, Hospital Anxiety and Depression Scale-subscale Anxiety; HADS-D, Hospital Anxiety and Depression Scale-subscale Depression; CFQ, Cognitive Failures Questionnaire; PSQI, Pittsburgh Sleep Quality Index; USER-P, Utrecht Scale for Evaluation of Rehabilitation-Participation; iPCQ, iMTA Productivity Cost Questionnaire; HRQoL, Health-Related Quality of Life; EQ-5D-5L, 5-level EuroQoL-5D; EQ-VAS, EQ-Visual Analogue Scale; SF-36, 36-item Short Form Health Survey.

**Table S6.** Comparison of baseline characteristics at hospital admission between participants with and without PEM at 3 years post-discharge.

|  | **Participants with PEM**  **(N=105)** | **Participants without PEM**  **(N=187)** | **P value** |
| --- | --- | --- | --- |
| **Patient characteristics** |  |  |  |
| Age, years | 60 (54─67) | 63 (56─69) | 0.07 |
| Sex, male | 55 (52%) | 152 (81%) | **<0.001** |
| BMI, kg/m² | 29 (27─33) | 27 (25─31) | **0.006** |
| *Migration Background* |  |  | 0.16 |
| European | 86 (83%) | 167(89%) |  |
| Dutch Caribbean | 10 (10%) | 13 (7%) |  |
| Asian | 4 (4%) | 5 (3%) |  |
| Turkish | 3 (3%) | 1 (1%) |  |
| (North) African | 0 (0%) | 1 (1%) |  |
| *Education* |  |  | 0.05 |
| Low | 32 (31%) | 52 (28%) |  |
| Middle | 43 (42%) | 59 (32%) |  |
| High | 27 (27%) | 76 (41%) |  |
| *Employment* |  |  | **0.02** |
| Unemployed | 18 (18%) | 17 (9%) |  |
| Employed | 66 (64%) | 111 (60%) |  |
| Retired | 19 (18%) | 58 (31%) |  |
| *Smoking status* |  |  | 0.65 |
| Never | 43 (42%) | 73 (39%) |  |
| Former | 57 (55%) | 111 (59%) |  |
| Current | 3 (3%) | 3 (2%) |  |
| *Physical activity level* ^b^ |  |  | **0.014** |
| Inactive | 20 (20%) | 10 (5%) |  |
| Light | 57 (55%) | 103 (55%) |  |
| Moderate | 24 (23%) | 58 (31%) |  |
| Vigorous | 2 (2%) | 16 (9%) |  |
| *Comorbidities* |  |  |  |
| ≥1 | 93 (89%) | 142 (76%) | **0.009** |
| Obesity (BMI≥30 kg/m²) | 50 (48%) | 60 (32%) | **0.009** |
| Diabetes | 17 (16%) | 22 (12%) | 0.29 |
| Cardiovascular disease/ hypertension | 37 (35%) | 68 (36%) | 0.85 |
| Pulmonary disease | 40 (38%) | 30 (16%) | **<0.001** |
| Renal disease | 14 (13%) | 15 (8%) | 0.15 |
| Gastrointestinal disease | 9 (9%) | 7 (4%) | 0.09 |
| Neuromuscular disease | 18 (17%) | 14 (8%) | **0.01** |
| Malignancy | 9 (9%) | 27 (14%) | 0.14 |
| Autoimmune/inflammatory disease | 12 (11%) | 20 (11%) | 0.85 |
| Mental disorder | 7 (7%) | 3 (2%) | **0.02** |
| **In-hospital characteristics** |  |  |  |
| *COVID-19 wave* |  |  | 0.74 |
| First | 37 (35%) | 62 (33%) |  |
| Second | 49 (47%) | 84 (45%) |  |
| Third | 19 (18%) | 41 (22%) |  |
| *COVID-19 directed treatment* |  |  |  |
| None | 5 (5%) | 4 (2%) | 0.07 |
| (Hydroxy)chloroquine | 5 (5%) | 4 (2%) | 0.21 |
| Steroids | 66 (63%) | 133 (71%) | 0.29 |
| Antivirals | 15 (14%) | 19 (10%) | 0.15 |
| Anti-inflammatory | 11 (11%) | 26 (14%) | 0.40 |
| Convalescent plasma | 1 (1%) | 4 (2%) | 0.45 |
| Thrombosis | 19 (18%) | 38 (20%) | 0.66 |
| Delirium | 31 (30%) | 49 (26%) | 0.84 |
| Requiring oxygen supplementation | 102 (97%) | 181 (97%) | 0.87 |
| Requiring high flow nasal cannula | 40 (38%) | 56 (30%) | 0.31 |
| ICU admission | 54 (51%) | 74 (40%) | 0.05 |
| Invasive mechanical ventilation | 50 (48%) | 67 (36%) | 0.05 |
| Length of intubation, days | 15 (8─30) | 17 (9─29) | 0.52 |
| Tracheostomy | 16 (15%) | 28 (15%) | 0.92 |
| Length of ICU stay, days | 18 (10─34) | 20 (10─35) | 0.70 |
| Length of hospital stay, days | 17 (8─34) | 13 (6─31) | 0.12 |

Data are presented as median (interquartile range) or n (%). The following variables were dichotomized for statistical analysis: migration background was categorized as European versus non-European groups combined, smoking status as never versus former/current, physical activity level as inactive/light versus moderate/vigorous, and treatment as no treatment versus any received treatment. P values are obtained using Mann-Whitney U test or Chi-squared test as appropriate, a P value less than 0.05 was considered statistically significant and is indicated in bold. PEM, Post-Exertional Malaise; BMI, Body Mass Index; ICU, Intensive Care Unit; NA, Not Applicable.

^a^ Number of patients with available data for each variables.

^b^ Pre-COVID-19 leisure time physical activity level was measured with the Saltin−Grimby Physical Activity Level Scale questionnaire.

**Table S7.** The frequency and severity of PEM symptoms in patients at 3 years after hospitalization for COVID-19.

|  | **Frequency** | | | | | | **Severity** | | | | |
| --- | --- | --- | --- | --- | --- | --- | --- | --- | --- | --- | --- |
|  | **N** | **None of the time** | **A little of the time** | **About half the time** | **Most of the time** | **All of the time** | **N** | **Mild** | **Moderate** | **Severe** | **Very severe** |
| Dead, heavy feeling after starting to exercise | 295 | 125 (42%) | 116 (39%) | 23 (8%) | 20 (7%) | 11 (4%) | 169 | 71 (42%) | 72 (43%) | 21 (12%) | 5 (3%) |
| Next day soreness or fatigue after non-strenuous, everyday activities | 293 | 106 (36%) | 111 (38%) | 28 (9%) | 37 (13%) | 11 (4%) | 187 | 73 (30%) | 76 (41%) | 35 (19%) | 3 (2%) |
| Mentally tired after the slightest effort | 292 | 125 (43%) | 112 (38%) | 22 (8%) | 27 (9%) | 6 (2%) | 166 | 73 (44%) | 74 (45%) | 17 (10%) | 2 (1%) |
| Minimum exercise makes you physically tired | 293 | 121 (41%) | 101 (34%) | 29 (10%) | 28 (10%) | 14 (5%) | 172 | 74 (43%) | 66 (38%) | 29 (17%) | 3 (2%) |
| Physically drained or sick after mild activity | 293 | 177 (60%) | 76 (26%) | 15 (5%) | 20 (7%) | 5 (2%) | 116 | 36 (31%) | 56 (48%) | 23 (20%) | 1 (1%) |

Post-exertional malaise (PEM) was assessed using a modified version of the DePaul Symptom Questionnaire. PEM was indicated if the frequency and severity of symptoms both were scored ≥2 for one or more symptoms. The frequency of each symptom was assessed as ‘*Throughout the past 6 months, how often have you had this symptom?*’. The severity of each symptom was assessed as ‘*Throughout the past 6 months, how much has this symptom bothered you?*’.
